# Transcripts expressed using a bicistronic vector pIREShyg2 are sensitized to nonsense-mediated mRNA decay

**DOI:** 10.1186/1471-2199-11-42

**Published:** 2010-06-01

**Authors:** Yayoi Shikama, Huiyuan Hu, Makiko Ohno, Isao Matsuoka, Tsutomu Shichishima, Junko Kimura

**Affiliations:** 1 Department of Pharmacology, Fukushima Medical University, Fukushima, Japan; 2 Department of Pharmaceutical Toxicology, School of Pharmaceutical Sciences, China Medical University, Shenyang, China; 3 The Laboratory of Pharmacology, Faculty of Pharmacy, Takasaki University of Health and Welfare, Takasaki, Japan; 4 Department of Cardiology and Hematology, Fukushima Medical University, Fukushima, Japan; 5 Fukushima Research Institute of Environment and Medicine, Futaba, Japan

## Abstract

**Background:**

pIREShyg2 has been widely used as a bicistronic expression vector. However, it is not known if the vector would affect the expression of cloned genes via nonsense-mediated mRNA decay (NMD), an mRNA surveillance system that degrades mRNA with a premature termination codon (PTC). In mammalian cells, the induction of NMD requires either a long 3'UTR or the presence of an exon-junction complex downstream of a PTC. The efficiency of NMD is greater when a PTC generates longer 3'UTR. pIREShyg2 provides the first cistron gene with a long 3'UTR consisting of a downstream intervening sequence (IVS), an internal ribosomal entry site (IRES) and the second cistron. Therefore, we hypothesized that the first cistron genes in pIREShyg2 are sensitized to NMD, which affects their expression levels. To examine this hypothesis, cDNAs encoding human granulocyte-macrophage colony-stimulating factor receptor β chain (βc) and its splice variant (βc79), in which the retention of a 79-base intron caused a frameshift generating 18 PTCs, were cloned into pIREShyg2 and stably expressed in a murine cell line, Ba/F3.

**Results:**

Compared with wild-type βc, the mRNA levels of βc79 were less than one tenth and decayed faster. Both translation inhibition and Upf1 knockdown led to significantly greater up-regulation of βc79 than wild-type βc. However, the use of a monocistronic pMT21 vector abolished the up-regulatory effects of translation inhibition and Upf1 knockdown on both wild-type βc and βc79, suggesting that the NMD is attributable to a structural determinant in pIREShyg2. The elimination of the intron and the proximal 3' 17 PTCs did not alter the greater effects of translation inhibition on βc79, suggesting that the first PTC, which determines 3'UTR length, was sufficient to enhance NMD efficiency. Thus, transcripts of PTC-harboring genes with longer 3'UTR are more efficiently degraded by the vector-dependent NMD than those of wild-type genes with relatively shorter 3'UTR, resulting in minimized expression of truncated mutants.

**Conclusions:**

We conclude that pIREShyg2, which sensitizes its bicistronic transcripts to NMD, may be useful for studying NMD but should be avoided when maximum expressions of PTC-harboring genes are required.

## Background

Expression vectors containing an internal ribosome entry site (IRES) element have been widely used as bicistronic vectors that provide co-expression of two unrelated reading frames from a single transcript unit [1-6]. A reading frame in a multiple cloning site downstream of a promoter is called the first cistron, and the second cistron is downstream of an IRES element. pIREShyg2 is a bicistronic expression vector that possesses an intervening sequence (IVS) between the first cistron and an IRES element derived from encephalomyocarditis virus, and a hygromycin resistance gene in the second cistron, which serves as a selection marker for stable transfection. It has been shown that the first cistron gene is expressed at levels comparable to those achieved in a monocistronic vector and initiation of translation is cap-dependent [7]. However, the present study is the first to show that the use of pIREShyg2 affects the mRNA stability of their carrying genes in mammalian cells, potentially leading to their insufficient expression.

Nonsense-mediated mRNA decay (NMD) is a post-transcriptional mRNA quality control system that eliminates aberrant mRNAs harboring premature termination codons (PTCs) within protein coding regions in eukaryotes [8-10] to protect the cells from accumulation of harmful or nonfunctional C-terminally truncated polypeptides [11,12]. The degradation occurs in a translation-dependent manner when translation is initiated in an mRNA cap-dependent manner [13,14]. In mammalian cells, two determinants have been identified that distinguish "premature" termination codons from "normal" termination codons and provide a protective advantage to the normal termination codon [15]. One is the presence of an exon-junction complex (EJC) more than 50 nucleotides downstream of a termination codon [16-23]. Induction of NMD requires the association between the EJC and the protein complex bound to the ribosome stalled at a PTC, which contains essential proteins to trigger NMD such as Upf1, eukaryotic release factors, and SMG1 [13,24-28]. Because normal termination codons generally reside either in the final exon or within 50 nucleotides upstream of the 3'-end in the penultimate exon, the transcripts coding wild-type proteins are able to escape NMD [16,29]. Another determinant is the distance between the stop codon and a poly(A) region [30-33]. Normal termination requires the interaction between the terminating ribosomal complex and the poly(A)-binding proteins (PABP), which leads to faster release of a terminating ribosome from mRNA [34]. A ribosomal complex at a PTC fails to interact with PABPs because of the relatively longer distance from the poly(A) region, resulting in prolonged association with mRNA, which stimulates NMD [28]. Recently, it has been reported that the length between a termination codon and poly(A) region affects NMD efficiency, showing that longer 3'UTR induces greater NMD activity [30-32].

Recently, we identified a novel splice variant of the granulocyte-macrophage colony-stimulating factor receptor (GMR) β chain (βc) in patients with myelodysplastic syndrome, a clonal hematopoietic disorder [35]. The splice variant (βc79) retained the 79-base intron V, resulting in a frameshift that introduced eighteen PTCs downstream of the retained intron. When the cDNAs encoding the βc79 or wild-type βc were cloned into the first cistron of pIREShyg2 vector and stably expressed in a murine hematopoietic cell line, Ba/F3, βc79 mRNA levels were significantly reduced compared with those observed for wild-type βc. We show here that the pIREShyg2 vector sensitizes the first cistron genes to NMD and, interestingly, that βc79 with PTCs was far more sensitive to the vector-dependent NMD resulting in minimized expression levels of the PTC-harboring gene.

## Results

### Intron V is not spliced out in βc79 clones

**Figure 1:**
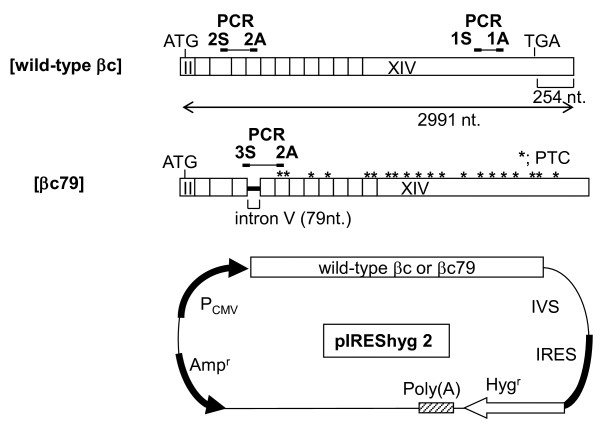
**Schematic presentation of wild-type βc- and βc79-constructs and positions of PCR primers**. βc cDNA from exon II to exon XIV, which contains the full length of the protein coding region, was inserted into a pIREShyg2 vector that had an IVS, an internal ribosome entry site (IRES), and hygromycin resistance gene between its cloning site and poly(A) region. The distance from the normal stop codon in wild-type βc to the IVS was 254 nucleotides. The retention of 79-base intron V in βc79 caused a frameshift that generated 18 PTCs. The first termination codon in βc79 was "TGA". Hyg^r^: hygromycin resistance gene, P_CMV_: human cytomegalovirus major immediate early promoter/enhancer.

**Figure 2:**
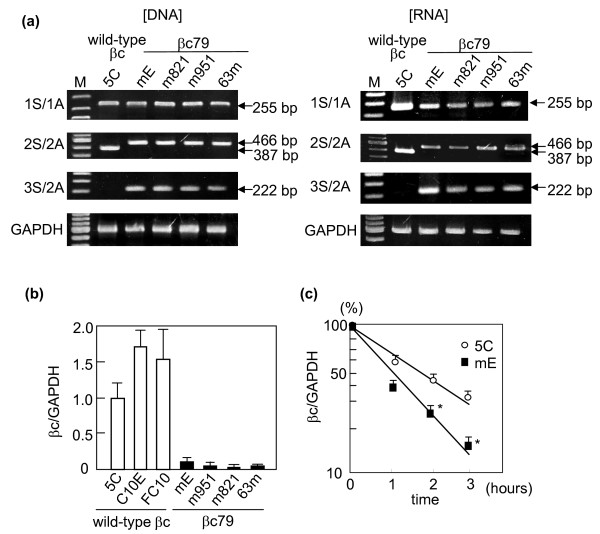
**DNA and RNA expression of introduced bc**. (a) DNA and RNA were isolated from a wild-type βc-transfected clone 5C and four βc79-transfected clones mE, m821, m951, and 63m. The isolated DNA and reverse-transcribed RNA were subjected to the amplification of βc and GAPDH by PCR. Primers 1S/1A, 2S/2A, and 3S/2A were used for detection of βc. cDNA obtained from 5C was diluted by 10 times, while cDNA from βc79-expressing clones were directly applied to PCR without dilution. M: Marker. (b) The βc-specific transcripts were quantified by real-time PCR using primer 1S/1A. The amounts of βc-specific transcripts relative to those of endogenous GAPDH transcripts were compared among three wild-type and four βc79 clones. The average values from 3 runs of real-time RT-PCR are presented. Error bars express S.E.M. (c) The mE and 5C cells were cultured in the presence of 25 μg/mL DRB and the ratios of βc-specific transcripts to GAPDH transcripts were plotted at various time points. *: significantly lower than the ratio from 5C cells at the same time points (p < 0.05).

To determine whether the intron V was spliced in the cells stably expressing βc79, we analyzed the presence of intron V in the integrated DNA and respective RNA isolated from each clone by PCR. The locations of the sequences corresponding to the primers are shown in Figure 1 and Table 1. PCR with 2S/2A primers that flank intron V distinguished βc79 cDNA from wild-type βc cDNA by the larger fragment size (446 bp versus 387 bp) (Figure 2a). In RNA from all βc79 clones, only the larger size of PCR products was detectable. The absence of a 387-bp band suggested that intron V in βc79 mRNA was not spliced out in Ba/F3 transfectants resulting in the generation of 18 PTCs. The unspliced intron V in βc79 mRNA was also detected by PCR using a 3S primer that was complementary to the 5'-end of intron V. All RNA samples without reverse transcription failed to generate any PCR products (data not shown).

**Table 1:** Sequences of primers.

Primer	Sequence (5'→ 3')	Nucleotide position
1S	TACCTGTGTCTGCCTGCTG	1910-1928 in βc
1A	GAGACATAACCAGAGGCCACT	2136-2166 in βc
2S	TGCCAGAGTTTTGTCGTCAC	326-345 in βc
2A	CTTGCTGGGACGTCCTGAGA	692-712 in βc
3S	CTTGGGAGGTAGGAACCACG	570-(i)12* in βc
HygRS	TGGATATGTCCTGCGGGTAA	2073-2092 in IREShyg2
HygRA	TTCCTTGCGGTCCGAATG	2345-2362 in IREShyg2
EpoRS	CCACTGCTTACTGGCTTATCG	838-859 in IREShyg2
EpoRA	TGCTCTCAAACTTGGGGTCCG	119-139 in EpoR

*(i), the nucleotide position in the intron V-derived sequence

### βc79 mRNA decays faster than wild-type βc mRNA

We determined the amounts of integrated DNA and respective transcripts for wild-type βc or βc79 by real-time PCR with 1S/1A primers. Compared with a clone expressing wild-type βc 5C, βc79 clones mE and m821 had similar amounts of integrated βc79 cDNA, and about half of that amount was found in clones m951 and 63m (data not shown). However, as shown in Figure 2b, in all four βc79 clones, RNA levels for βc79 were less than 10% of that measured for wild-type βc mRNA in 5C. Two other independent clones of wild-type βc, C10E and FC10, showed higher mRNA levels than 5C. We next added 5,6-dichlorobenzimidazolen1-β-D-ribofuranoside (DRB), a transcription inhibitor, to mE and 5C cells and determined the steady-state amounts of wild-type βc and βc79 transcripts over time. Wild-type transcripts (relative to GAPDH) decreased to 50% of the initial level after two hours, while βc79 transcripts (relative to GAPDH) in mE fell to half the initial level after one hour (Figure 2c). The reduction of βc79 transcripts was significantly greater than that of wild-type βc at the two and three hour time points. Given that intron V was not spliced out in Ba/F3 transfectants, we hypothesized that the presence of 18 PTCs in βc79 mRNA upstream of the pIREShyg2-derived IVS resulted in NMD induction.

### Upregulation of βc79 transcripts by a translation inhibitor is greater than that of wild-type βc

**Figure 3:**
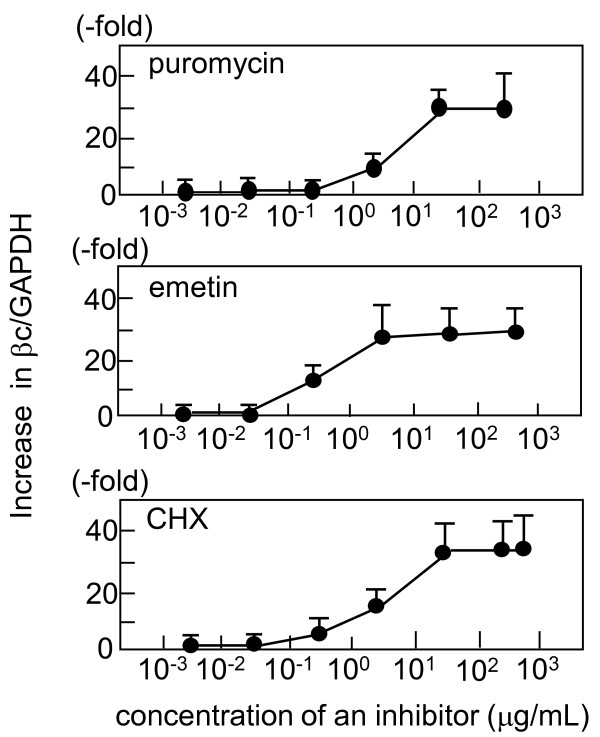
**Dose-response relationship between βc79 transcripts and translation inhibitors**. The βc79-expressing clone mE was incubated with various concentrations of puromycin, emetin, and CHX for 4.5 hours. The ratios of βc-specific transcripts to GAPDH transcripts were measured by real-time RT-PCR with 1S/1A primers. The ratios in NMD inhibitor-treated cells were divided by those in untreated cells, which are presented as fold increases. The mean values ± S.E.M. (error bars) were obtained from 3 to 4 independent experiments.

It was shown that the treatment of cells with translation inhibitors result in abrogation of NMD that occurs in a translation-dependent manner [36,37]. Therefore, to examine whether NMD is implicated in the degradation of βc79 mRNA, mE cells were incubated with various concentrations of puromycin, a translation inhibitor. The amounts of βc79 transcripts relative to those of GAPDH increased in a dose-dependent manner, and the maximum level (30.3 ± 1.4-fold) was observed with 20 μg/mL puromycin (Figure 3). Emetin and cycloheximide (CHX), two other inhibitors, also induced a dose-dependent increase in βc79 transcripts and maximum effects similar to those induced by puromycin (31.0 ± 7.0-fold, and 35.0 ± 1.4-fold, respectively) (Figure 3).

**Figure 4:**
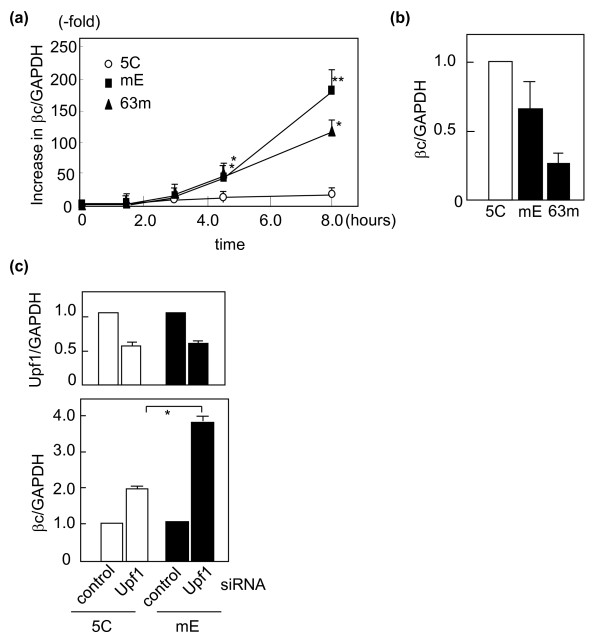
**Time course of βc transcript levels in response to puromycin**. (a) Two βc79 clones, mE and 63m, and a wild-type βc clone 5C were incubated with 100 μg/mL puromycin for the indicated periods, and the ratios of βc-specific transcripts to endogenous GAPDH transcripts were measured by real-time RT-PCR with 1S/1A primers. The values at 0 hours are plotted as 1.0. The average values ± S.E.M. (error bars) were obtained from 3 to 8 experiments. * and **: significantly higher than the value in 5C incubated for the same time with p < 0.05 and p < 0.01, respectively. (b) After 8-hour treatment, the βc-specific transcripts were quantified and normalized by those of GAPDH. The values from mE and 63m cells were compared with those from 5C cells, which are plotted as 1.0. (c) The siRNA targeting Upf1 was introduced into 5C and mE cells, and the amounts of Upf1 and βc transcripts were quantified after 48 hours. The presented data are the average values and S. E. M. (error bars) f3rom four different experiments. *: significantly different (p < 0.05).

To compare the effects of the translation inhibitor between βc79 and wild-type βc, mE and 63m clones expressing βc79 and clone 5C expressing wild-type βc were incubated with 100 μg/mL puromycin, and βc-specific transcripts were quantified at various time points (Figure 4a). A time-dependent increase in βc-specific transcripts was observed in all three clones. At 4.5 hour time point, the increase of βc79 transcripts in both mE (41.4 ± 6.2-fold, p < 0.05) and 63m (39.6 ± 5.4-fold, p < 0.05) became significantly greater than that of wild-type transcripts in 5C (11.4 ± 1.3-fold). The different up-regulatory response between βc79 and wild-type βc was confirmed in two other clones of βc79 and wild-type βc (Table 2). Among four βc79-expressing clones, there was no significant difference in the increase of βc79 transcripts induced by puromycin. After treatment for 8 hours, the βc79 mRNA levels in mE and 63m further increased to 184.3 ± 27.9-fold and 115.7 ± 8.0-fold, respectively. In contrast, there was only an 11.9 ± 2.9-fold increase in wild-type βc transcripts in 5C. The resulting mRNA levels of βc79 in mE and 63m became 66% and 26% of wild-type βc, respectively (Figure 4b).

**Table 2:** Effects of incubation with 100 μg/ml puromycin for 4.5 hours.

Wild-type βc clones	Increase (-fold)	βc79 clones	Increase (-fold)
5C	11.4 ± 1.3	mE	41.4 ± 6.2
C10E	9.6 ± 1.5	63 m	39.6 ± 5.4
FC10	7.3 ± 0.7	m821	33.3 ± 1.2
		m951	49.6 ± 3.2

Presented as average values and S.E.M. from three to eight independent experiments

To confirm that the effects of NMD inhibition were greater on βc79 than on wild-type βc, siRNA targeting Upf1, an essential component of the NMD machinery, was used in mE and 5C cells. In both Upf1 siRNA-treated mE and 5C cells, the Upf1 RNA levels became 25-30% those of the control siRNA-treated cells after 24 hours, and 50-55% after 48 hours. As shown in Figure 4c, the reduction in Upf1 for 48 hours resulted in a 3.5 ± 0.4-fold up-regulation of βc79 transcripts in mE, which was significantly greater than that of wild-type βc in 5C (1.9 ± 0.2-fold, p < 0.05).

### Vector sensitizes both wild-type βc and βc79 to NMD

**Figure 5:**
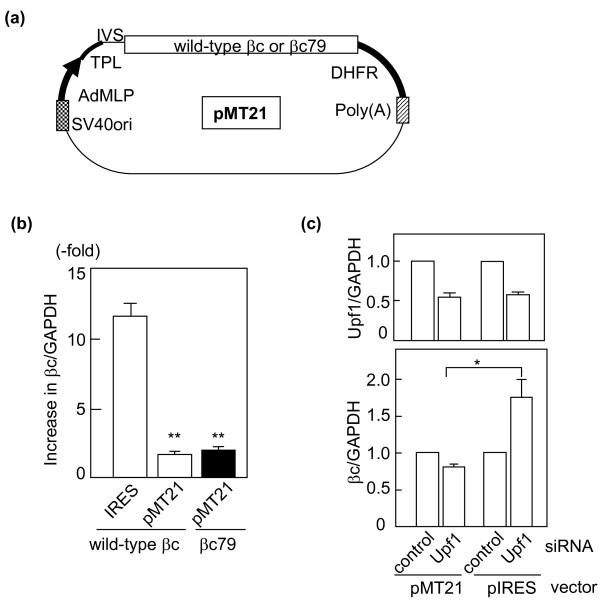
**Effects of puromycin and Upf1 knockdown on the constructs cloned in a pMT21 vector**. (a) The structure of the pMT21 vector. SV40ori: simian virus 40 (SV40) origin of replication and enhancer element; AdMLP: adenovirus major late promoter; TPL: the tripartite leader; IVS: intervening sequence; DHFR: murine dihydrofolate reductase coding region; poly(A), polyadenylation signal. (b) The cDNAs of wild-type βc and βc79 were stably expressed in Ba/F3 cells using a pMT21 vector. These transfectants and 5C cells that expressed the wild-type βc in pIREShyg2 were incubated with or without 100 μg/mL puromycin for 4.5 hours. The values for βc/GAPDH in puromycin-treated cells were divided by the values in untreated cells, which are presented as fold increases of βc/GAPDH. Data shown are the average values ± S.E.M. (error bars) (pIREShyg2: n = 8, pMT21: n = 3). **: significantly smaller than the value in the wild-type βc cloned into pIREShyg2 (p < 0.01). (c) Upf1 was knocked down by siRNA in the cells expressing wild-type βc in pIREShyg2 (5C) and pMT21. The amounts of Upf1 and βc transcripts relative to GAPDH from three independent experiments are presented. *: significantly different (p < 0.05).

Although wild-type βc transcripts were less affected by NMD inhibition than βc79 transcripts, treatment with puromycin always resulted in a 7 to 12-fold increase in the amount of wild-type βc transcripts in Ba/F3 cells. Interestingly, pIREShyg2 provides an IVS, an IRES and a hygromycin resistance gene downstream of the normal termination codon in wild-type βc. We wondered whether the structural characteristics of pIREShyg2 might cause NMD in wild-type βc. To test this hypothesis, cDNA of the wild-type βc was cloned into a monocistronic pMT21 vector that did not have them between its cloning site and poly(A) region (Figure 5a), and stably expressed in Ba/F3 cells. In contrast to what we observed when βc was cloned into pIREShyg2 (11.4 ± 1.3-fold increase), transcripts of wild-type βc cloned into pMT21 showed only a slight increase (1.4 ± 0.2-fold) after incubation with puromycin for 4.5 hours. This difference was highly significant (p < 0.01) (Figure 5b). The siRNA-mediated Upf1 knockdown also resulted in a 0.81 ± 0.02-fold change in the wild-type βc in pMT21, which was significantly lower than that in pIREShyg2 (1.8 ± 0.2-fold, p < 0.05) (Figure 5c). When βc79 was cloned into pMT21 instead of pIREShyg2, the up-regulatory response of βc79 diminished to 1.9 ± 0.2-fold, which was not significantly different from that of wild-type βc cloned into pMT21 (Figure 5b).

We next examined whether pIREShyg2-dependent NMD affected the expression of the second cistron. The transcripts of hygromycin resistance gene (HygR) in the cells expressing wild-type βc was increased 8.7 ± 1.6-fold (n = 4) by puromycin treatment for 4.5 hours. The increase of HygR transcripts in βc79 clones was 39.7 ± 1.3-fold (n = 3), which was significantly greater than that in wild-type cells (P < 0.05).

### Replacement of intron V with a single nucleotide or elimination of the intron preserving a 5' single PTC fail to alter NMD effects on βc79

**Figure 6:**
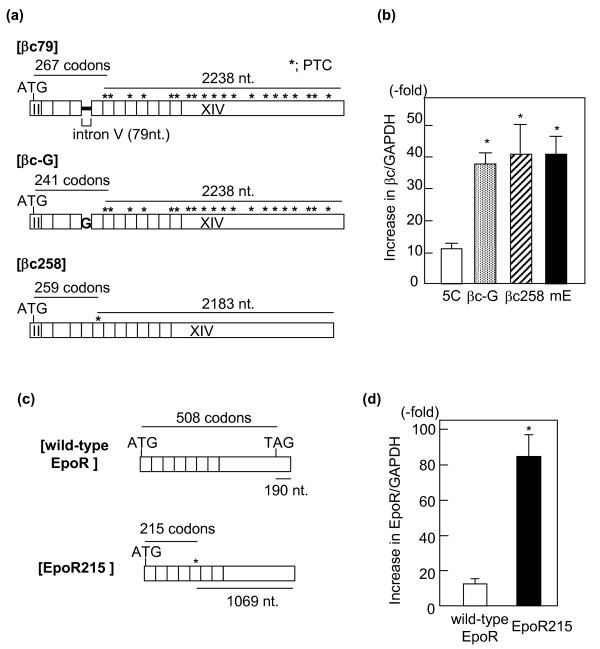
**Replacement of the intron V with a single nucleotide**. (a) βc-G was constructed by replacing the intron V-derived 79 nucleotides with a single nucleotide G in βc79. The frameshift generating eighteen PTCs were preserved in βc-G. βc258 was a βc with a single PTC at the 259 th codon. Both βc79 and βc-G had 2238 nucleotides downstream of their termination codon, and βc258 had 2183 nucleotides. (b) βc-G and βc258 were cloned into a pIREShyg2 vector and stably expressed in Ba/F3 cells. The cells expressing βc-G, βc258, βc79 (mE) and wild-type βc (5C) were cultured with or without 100 μg/mL puromycin for 4.5 hours. The amounts of βc-specific transcripts relative to GAPDH transcripts in puromycin-treated cells were divided by the amounts in untreated cells, and are presented as-fold increases of βc/GAPDH. Average values and S.E.M. (error bars) were obtained from three independent experiments. * significantly higher than 5C (p < 0.05). (c) EpoR215 was an EpoR with a termination codon (TAG) at the 215 th codon generating 1099 nucleotides downstram of the termination codon. In wild-type EpoR, there were 190 nucleotides downstream of its normal termination codon. (d) Wild-type EpoR and EpoR215 were stably expressed in Ba/F3 cells using a pIREShyg2 vector, and cultured with or without 100 μg/ml puromycin for 4.5 hours. The ratios of EpoR-specific transcripts to GAPDH transcripts in puromycin-treated cells were divided by those in untreated cells. Average values and S. E. M. (error bars) were obtained from three different experiments. * significantly higher than the value in wild-type EpoR (P < 0.05).

To examine whether the greater NMD activity on βc79 was attributable to intron V retained in βc79, the 79 nucleotides of intron V were replaced by a single guanylate residue as indicated by "G" in Figure 6a. Although the 78 nucleotides of the intronic sequence had been deleted in the resulting cDNA (βc-G), βc-G still retained the same 18 PTCs as βc79 (Figure 6a). βc-G was cloned into IREShyg2 and stably expressed in Ba/F3 cells. As shown in Figure 6b, puromycin treatment for 4.5 hours increased βc-G transcripts 38.6 ± 2.0-fold, which was significantly higher than the increase in 5C (p < 0.05) but not different from that in mE (41.4 ± 5.4-fold). This result suggests that the presence of intron V per se is not directly involved in mediating greater NMD on βc79 than on wild-type βc. We next wanted to test whether preservation of the first PTC mutation (position 259) in a variant of βc79 in which intron V had been completely deleted still conferred NMD to the expressed transcripts (Figure 6a). The resulting cDNA, βc258, was stably expressed using pIREShyg2. The up-regulatory effect of puromycin on βc258 (41.6 ± 10.9-fold) was not significantly different from that on βc79 (Figure 6b).

Lastly, we examined whether the pIREShyg2-dependent NMD was specific to βc. cDNAs of the wild type and a truncated form (EpoR215) of mouse erythropoietin receptor (EpoR), which are shown in Figure 6c, were subcloned into pIREShyg2 and stably expressed in Ba/F3. The puromycin treatment for 4.5 hours increased wild-type EpoR transcripts 12.8 ± 1.9-fold. In contrast, EpoR215 was increased 84.8 ± 16.5-fold, which was significantly greater than that of wild type (p < 0.05). (Figure 6d).

## Discussion

This study reveals that an expression vector can destabilize the mRNA of its carrying gene via NMD resulting in minimized expression levels.

When βc79 and wild-type βc were stably expressed using a commercial expression vector, pIREShyg2, the mRNA levels of βc79 were less than one tenth those of wild-type βc. mRNA expression levels are regulated through transcriptional and posttranscriptional control mechanisms [36-38], and mRNA stability is determined by posttranscriptional regulation, which varies from one mRNA species to another [39,40]. The diversity in the transcriptional activities can be due to their integration sites [41,42]. However, the low βc79 expression was uniformly seen in all clones tested, and the transcripts of βc79 decayed faster than those of wild-type βc. These indicate that the posttranscriptional mechanism was involved in the low expression levels of βc79 mRNA. The RT-PCR indicated that 79-base intron V was retained in the βc79 transcripts, generating 18 PTCs and longer 3' UTR in βc79 than in wild-type. Since transcripts with longer 3'UTR are more sensitive to NMD in mammalian cells [30-33], we examined whether destabilization of βc79 mRNA via NMD caused the suppression of βc79 expression levels.

The faster decay of βc79 mRNA is attributable to greater effects of NMD on βc79 than on wild-type βc. Since NMD is a translation-dependent process, translation inhibitors have been used to abrogate NMD, and the increase in mRNA levels in the presence of a translation inhibitor is considered to reflect the effects of NMD [43,44]. The up-regulation of βc79 transcripts by three different translation inhibitors indicated that βc79 mRNA was degraded by NMD. Furthermore, the up-regulatory effects of the translation inhibition were significantly greater on βc79 than on wild-type βc, which diminished the difference in mRNA levels between βc79 and wild-type βc. The greater effects of NMD on βc79 were also confirmed by siRNA-mediated NMD inhibition. Transcripts of βc79 were thus degraded more efficiently by NMD than those of wild-type βc, which resulted in lower mRNA levels of βc79.

Wild-type βc was also sensitive to NMD when expressed by pIREShyg2, because both translation inhibition and Upf1 knockdown increased transcripts of wild-type βc cloned into pIREShyg2. These up-regulatory effects were abrogated when pMT21 was used instead of pIREShyg2. This observation indicates that the vector sensitized wild-type βc to NMD. Because βc79 also became insensitive to NMD in pMT21, we concluded that the IRES vector rendered its first cistronic genes sensitive to NMD.

There may be additional determinants for the stronger NMD to βc79 in the pIREShyg2 system. Although the nucleotide in the position immediately downstream of the termination codon was shown to enhance the up-regulatory effects of translation inhibition in the order G < U, A < C [45,46], it does not seem relevant to the difference between βc79 and the wild-type βc, because the nucleotide following the first PTC in βc79 and the one after the normal stop codon in wild-type βc are both G. The replacement of the intron V with a single nucleotide did not alter the up-regulatory effects of NMD inhibition, suggesting that the intron V was not responsible for greater NMD effects on βc79. The βc258, which had a single PTC at a position similar to the distal 5' PTC in βc79 but no frameshift, elicited a degree of NMD similar to that of βc79, indicating that the 5' single PTC is sufficient to induce greater NMD effects on βc79. This is comparable to recent reports in which more efficient NMD was induced when a PTC was generated closer to 5'-end resulting in longer 3'UTR length [30-32].

Lastly, it is unlikely that pIREShyg2-related NMD induction is specific to βc cDNA. When wild-type EpoR was stably expressed in the same cell line using pIREShyg2, upregulation of its transcripts was induced by puromycin, suggesting that wild-type EpoR in pIREShyg2 vector was also a target of NMD. The effects of puromycin were enhanced on EpoR215 that possessed longer 3'UTR.

Thus, the expression levels of genes encoding truncated mutants, in which PTCs generate longer 3'UTRs, can be significantly reduced via vector-dependent NMD compared with wild-type genes that generally have shorter 3'UTR.

## Conclusions

We show here for the first time the NMD effects associated with an expression vector in a mouse hematopoietic cell line. Although the IRES vector may be an interesting system for studying NMD, our study warns us that the choice of the IRES vector leads to undesirable consequences especially when maximum expression of a truncated mutant with a long 3'UTR is required in mammalian cells.

## Methods

### Plasmid constructions

The cDNA of full-length GM-CSF receptor βc subunit (kindly provided by Dr. Sumiko Watanabe, University of Tokyo, Tokyo, Japan) was subcloned into the SmaI site of pIREShyg2, an expression vector (Clontech Laboratories, Inc., Palo Alto, USA), as shown in Figure 1. The βc79 cDNA containing intron V at nucleotide position 578 was isolated from a patient with myelodysplastic syndromes and subcloned into a plasmid vector, pCR4-TOPO (Invitrogen Corp, Carlsbad, USA) to confirm its sequence. The Acc I site to BssH II fragment of βc79, which contains intron V, was used to replace the Acc I-BssH II fragment of wild-type βc in pIREShyg2, thus generating the βc79-IREShyg2 construct.

Wild-type βc in pMT21 was obtained using the construct of the EpoR-βc hybrid receptor in pMT21, which was prepared in a previous study [47]. The hybrid receptor consisted of 756 bases proximal to 5' of EpoR cDNA and 1655 bases proximal to 3' of βc cDNA. The fragment between EcoRI and BssHII sites of the hybrid receptor in pMT21, which contained the EpoR-coding region, was substituted to cDNA of wild-type βc or βc79 in pcDNA3.1 that possessed an EcoRI site in the multiple cloning region. The obtained full-length βc cDNA in pMT21was sequenced prior to transfection.

cDNA of murine EpoR, a generous gift from Dr. Alan D'Andrea (Dana-Farber Cancer Institute, Boston, USA), was inserted between Eco47III and EcoRV sites in pIREShyg2 vector.

The constructs harboring a single PTC were generated using QuickCHange II XL Site-Directed Mutagenesis Kit (Stratagen-An Agilent Technologies Company, La Jolla, USA), and sequenced prior to transfection.

### Transfection

The constructs were introduced into Ba/F3 cells, an IL-3-dependent mouse hematopoietic progenitor cell line, as described previously [35]. The hygromycin-resistant cells were subjected to repeated limiting dilutions to establish independent clones.

### Inhibitions of transcription and translation

Cells (grown at 1 × 10^6 ^cells/ml) were cultured in the presence of 25 μg/mL DRB (Sigma-Aldrich Corp., St. Louis, MO), a transcription inhibitor, or the indicated concentrations of translation inhibitors: emetin (Sigma), puromycin (Sigma), or CHX (Sigma).

### Upf1 knockdown by siRNA induction

The siRNA corresponding to mouse Upf1 mRNA was designed as follows: sense GAUGCAGUUCCGUUCCACUtt and antisense GAUGGAACGGAACUGCAUCtt (nucleotide 2084-2102; Genbank Accession No. AY597038). One and a half μg control siRNA (Ambion Inc., Austin, USA) or siRNA targeting to Upf1 was introduced into 0.2 × 10^5 ^cells suspended in 75 μL siPORT™ siRNA Electroporation Buffer (Ambion) by square-pulse electroporation (250 mV 30 msec) using a Gene Pulser (Bio-Rad Laboratories, Inc., Hercules, USA). After 24 and 48 hours, the transcripts of βc and Upf1 were quantified.

### Isolation of DNA and RNA

Genomic DNA was isolated from each clone using the Generation DNA purification system kit (Gentra Systems, Inc., Minneapolis, MN). Total cellular RNA was isolated by acid guanidium thiocyanate-phenol-chloroform extraction methods [48]. All RNA samples were treated with RNase-free DNaseI (Takara Bio, Inc., Otsu, Japan) prior to further analyses.

### PCR, and real-time PCR

The total cellular RNA was incubated in reverse-transcription buffer including random primer, dNTP, and RNAase inhibitor, with or without reverse transcriptase at 37°C for 1 hour as previously described [49], and subjected to PCR analyses. The annealing temperatures for detection of βc and others were 62°C and 56°C, respectively. The samples prepared without the enzyme were used to examine DNA contamination in the RNA samples. The sequences of PCR primers are presented in Table 1.

The βc-specific transcripts were quantified by real-time PCR in duplicate using SYBR Premix Ex Taq (Takara Bio. Inc.), 1S/1A primers (Table 1), and LightCycler (Roche Diagnostics GmbH, Mannheim, Germany). Glyceraldehyde-3-phosphate dehydrogenase (GAPDH) was also quantified as an internal control. The real-time PCR protocol included an initial denaturing step at 95°C for 30 seconds, 40 cycles of 5-second denaturing, 10-seccond annealing and 15-second extension, a cooling step to 65°C for 15 seconds followed by a heating step for dissociation analysis. The annealing temperatures for quantification of βc and others were 62°C and 56°C, respectively. Crossing points were determined by the second derivative maximum method. The amounts of targets were calculated based on standard curves which were generated by the amplification of sequentially diluted cDNA with each PCR primer pair. In every experiment, it was confirmed that the first derivatives of the dissociation curves had only a single peak.

### Statistical analysis

The comparison of data among more than three clones was done by Scheffe's test. The Mann-Whitney test was used for the comparison between two groups.

## Authors' contributions

YS conceived and designed the study. YS and HH quantified transcripts. YS, HH, and MO constructed a mutant cDNAs. IM and JK supervised the study design and data analyses. TS participated in the identification of the variant and the preparation of the manuscript. All authors read and approved the final manuscript.
